# Aortic isthmus massive calcifications: an unusual case of secondary hypertension

**DOI:** 10.1186/s13019-023-02390-3

**Published:** 2023-11-27

**Authors:** Sadia Soukeur, Benoit Cosset, Fadi Farhat

**Affiliations:** 1 Department of cardiology, Croix Rousse Hospital, Lyon, Bron, France; 2 Department of Cardiovascular Surgery B, Louis Pradel Hospital, Bron, France; 3 Department of Cardiovascular Surgery, Infirmerie Protestante, 1-3 chemin du Penthod, Caluire et Cuire, 69300 France

**Keywords:** Secondary hypertension, Aortic plaque, Pseudocoarctation, Heart failure

## Abstract

We present the case of a 63-year-old woman who was hospitalized five times in 4 months for episodes of heart failure, associated with a sustained hypertension despite a fivefold therapy. The pathophysiological mechanism of the hypertension was a secondary hyperaldosteronism linked to a renal hypoperfusion due to the narrowing of the thoracic aorta by a huge calcified plaque, mimicking an aortic coarctation.

## Background

Secondary hypertension is suspected when there is a severe elevation of blood pressure or a sudden onset or worsening of hypertension, hardly controlled by several drugs therapies. The classic causes are well known [1] and are easy to detect with a simple screening including interrogation, physical examination and routine laboratory investigations [2]. Sometimes, causes of secondary hypertension can be more atypical and harder to diagnose [3, 4].

Herein, we present an unusual case of secondary hypertension due to huge isthmic calcifications mimicking a coarctation syndrome.

### Case presentation

A 63-year-old woman with a past medical history of smoking and coronary heredity was referred to our institution because of refractory hypertension. In her medical history, we noted an abdominal aortic aneurysm and a lower limb arteriopathy.

One year before referral, she developed an oral Streptococcus septicemia associated to a first episode of cardiac insufficiency. After one month, she was hospitalized because of a lung oedema due to a hypertension access recurrence, followed by two others episodes of heart failure associated with hypertension despite a fivefold antihypertensive therapy (calcium channel blocker, beta blocker, central antihypertensive, potassium-sparing diuretic, loop diuretic). We noticed that each attempt of introduction of an ACE inhibitor or spironolactone was associated to a temporary acute renal insufficiency regressing after the withdrawal of these medications.

Medical examination made in a general hospital included a normal electrocardiogram. A transthoracic echography showed a mild dilated cardiomyopathy with aleft ventricular ejection fraction at 45%, a mild mitral valve regurgitation and a pulmonary artery hypertension.

Laboratory screening revealed elevated proBNP (41 000 pg/mL) and creatinine (125 µmol/L), and a hypokalemia (2.3 mmol/L). The values of urinary methoxyamines, as well as renin, were slightly elevated. The abdominal scanner made at the time of septicemia didn’t find adrenal gland abnormalities. An angiography showed a non-significant stenosis of one renal artery.

At arrival in our institution, the patient showed signs of left heart failure, systolic murmur in all auscultatory areas and abdominal murmur. There was a gradient between arms and legs blood pressures (mean of 50mmHg). A blood pressure holter confirmed the hypertension at the level of the right arm with an average pressure of 175/69 mmHg.

Blood tests confirmed the hypokalemia at 2.9 mmol/L, with normal creatinine and sodium levels. The urinary methoxyamines levels were raised: methoxytyramine at 2 N, normetanephrine at 3 N. Blood methoxyamines were subnormal. Aldosterone and renin were increased with a low aldosterone/renin ratio but a normal urinary free cortisol.

**Fig. 1:**
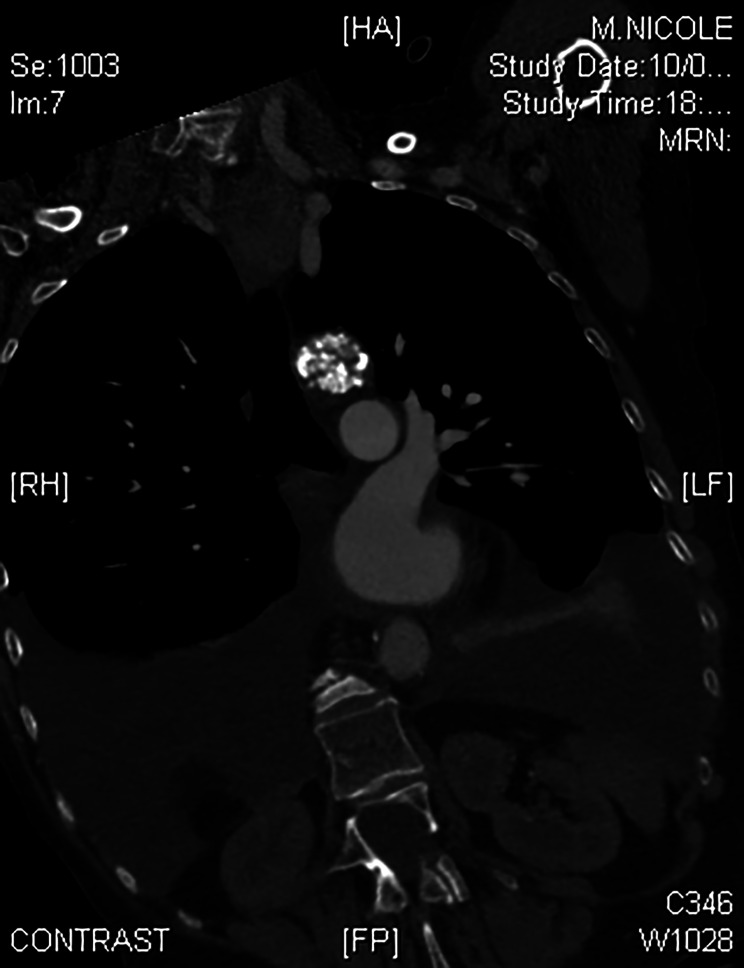
CT scan (frontal cut) showing massive calcifications in the aortic isthmus

**Fig. 2:**
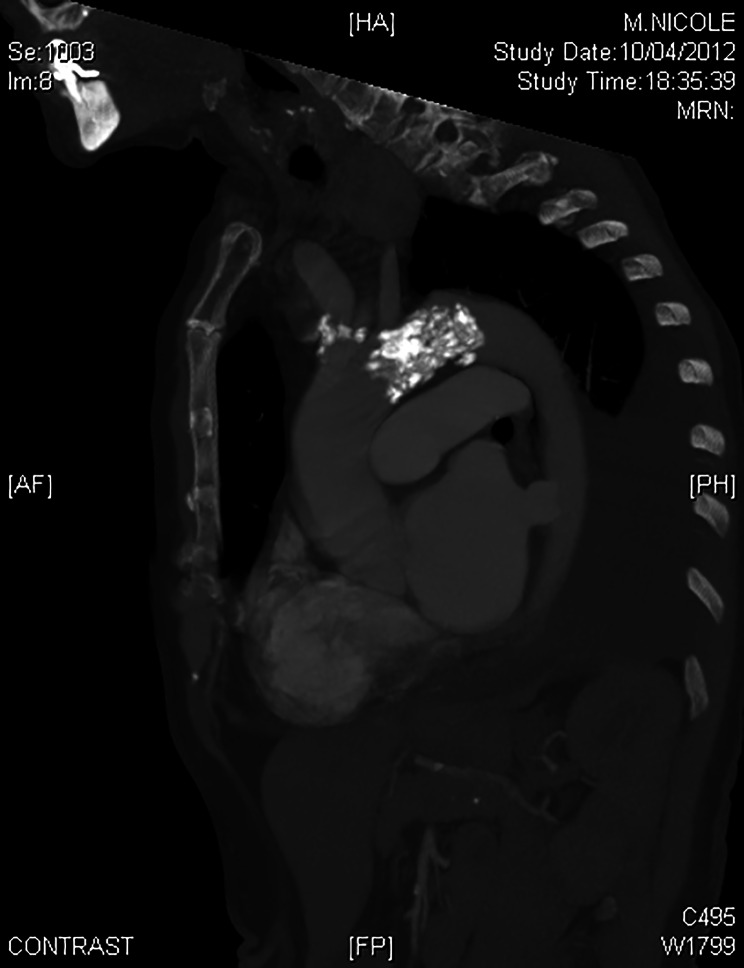
CT scan (sagittal cut) showing the calcified plaque rising up to the aortic arch in front of the left subclavian artery emergence

The renal arteries Doppler revealed a near occlusion of the upper portion of the abdominal aorta with a secondary hypoperfusion of mesenteric and celiac arteries. There was also a hypoperfusion of the renal parenchyma and arteries without stenosis. Therefore, a total aortic CT scan was performed. It showed huge calcifications of the aortic isthmus with a severe narrowing of the aortic lumen, mimicking a coarctation (Figs. 1 and 2). The calcifications extended to the origin of the left subclavian artery without obstruction of its ostium. The discussion between an endovascular or an open surgery was rapidly closed because of the risk of embolism and aortic rupture along with an endovascular treatment. Because of the extension of the calcifications towards the concavity of the aortic arch, a median sternotomy was decided to access the lesions.

**Fig. 3:**
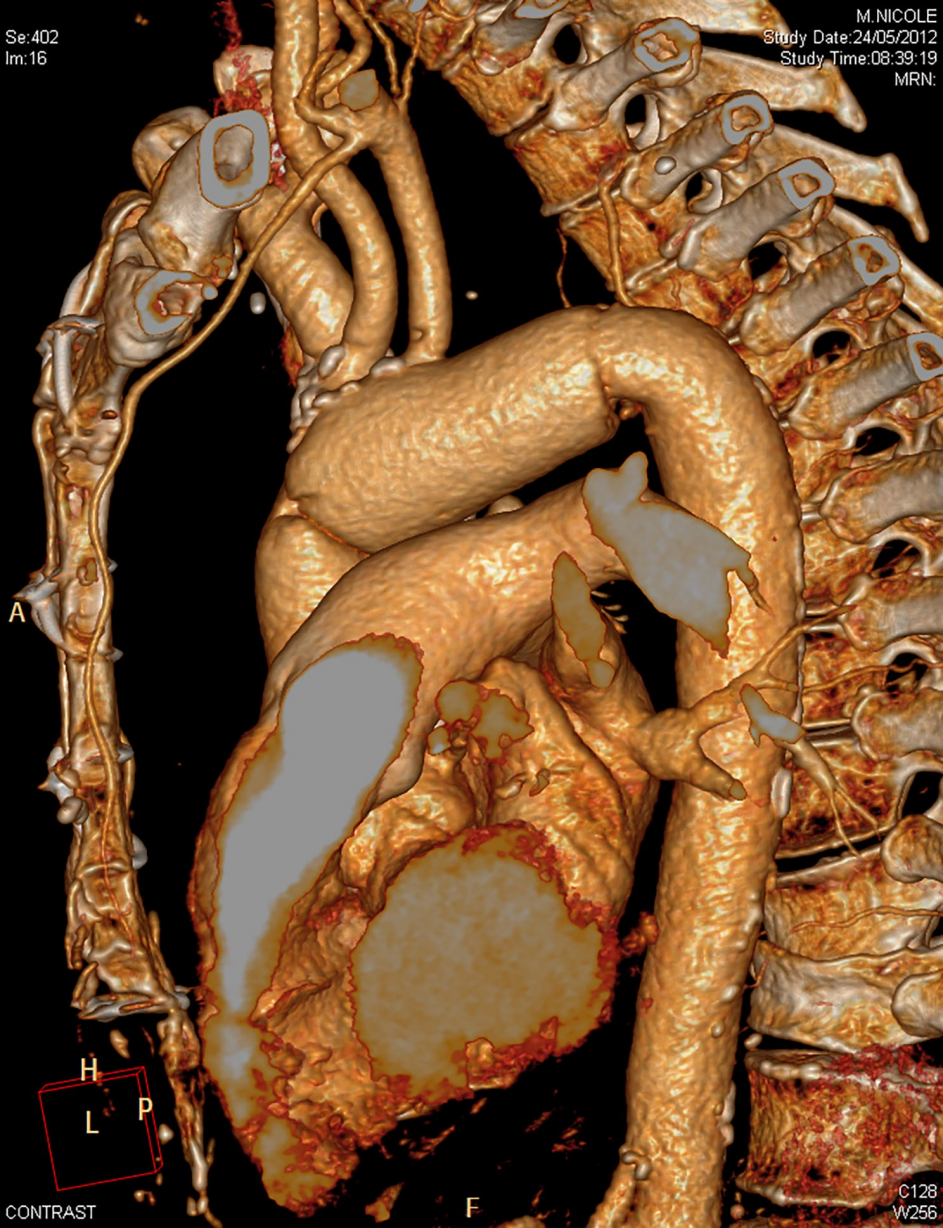
postoperative CT scan reconstruction with the replacement of the arch and isthmus by a straight Dacron tube

Surgery was made under cardiopulmonary bypass, peripheral circulatory arrest and selective antegrade cerebral perfusion at a body temperature of 28 °C. The whole arch and isthmus were resected and replaced using a 30 mm straight Dacron™ tube (Fig. 3). Since the patient was small shaped, the access to the isthmus was easy through the full sternotomy.

The post-operative course was uneventful, without other episode of heart failure and a well-balanced blood pressure. Bacteriological examination of the aortic specimen showed the same oral Streptococcus found during the septicaemia one year before. She was therefore considered as an infective endocarditis and treated in consequence. Further evolution showed a normalization of the blood pressure on 24-BP assessement at one and 6 months allowing the weaning of most of the antihypertensive therapies.

## Discussion

This case shows an original clinical presentation of a symptomatic resistant hypertension with refractory heart failure. The presence of another plaque at the level of the infra-renal aorta has confused the diagnosis. It is the combination of several factors including the data of the Doppler, which gave functional information, and the understanding of the pathophysiological mechanism that led to the second reading of the CT-scan and reoriented the diagnosis.

Indeed, the pathophysiological mechanism is the same as in the coarctation, which is a narrowing of the aortic lumen that causes a secondary hyperaldosteronism by a renal hypoperfusion. However, the shrinking of the aorta in this case is not due to a congenital malformation (0,2% of all secondary hypertension cases in young asymptomatic adults) but to a huge calcified plaque. This could be called a coarctation-like syndrome or a pseudo-coarctation. Acquired aortic calcifications are generally caused by aortitis such as Takayasu arteritis.

The other remarkable aspect is the presence in this lesion of the same Streptococcus responsible of the septicaemia a few months ago. It indicates that this lesion could have the same evolutive potential of an endocarditis. That’s why the patient was treated as an infective endocarditis.

## Data Availability

all data and images are available.
